# Clinical Factors Associated with Severity of Colonic Diverticular Bleeding and Impact of Bleeding Site

**DOI:** 10.3390/jcm12051826

**Published:** 2023-02-24

**Authors:** Hirohito Amano, Takatsugu Yamamoto, Ken Ikusaka, Naoaki Aoki, Miyoko Sakurai, Taku Honda, Kyohei Maruyama, Hitoshi Aoyagi, Akari Isono, Koichiro Abe, Yoshinari Asaoka, Shinya Kodashima, Atsushi Tanaka

**Affiliations:** 1 Department of Medicine, Teikyo University School of Medicine, Tokyo 173-8605, Japan; 2 Department of Gastroenterology, Nagoya Central Hospital, Nagoya 453-0801, Japan

**Keywords:** colonic diverticular bleeding, severity, invasive treatment, right colon

## Abstract

Factors associated with serious colonic diverticular bleeding (CDB) are unclear, although the incidence of CDB has increased. We carried out this study to clarify factors associated with serious CDB and rebleeding. Subjects included 329 consecutive patients hospitalized for confirmed or suspected CDB between 2004 and 2021. Patients were surveyed regarding backgrounds, treatment, and clinical course. Of 152 with confirmed CDB, 112 showed bleeding from the right colon, and 40 did from the left colon. Patients received red blood cell transfusions in 157 (47.7%), interventional radiology in 13 (4.0%), and surgery in 6 (1.8%) cases. Early rebleeding within one month occurred in 75 (22.8%) patients, and late rebleeding within one year occurred in 62 (18.8%). Factors associated with red blood cell transfusion included confirmed CDB, anticoagulants, and high shock index. The only factor related to interventional radiology or surgery was confirmed CDB, which was also associated with early rebleeding. Late rebleeding was associated with hypertension, chronic kidney disease and past CDB. Right CDB showed higher rates of transfusion and invasive treatment than left CDB. Confirmed CDB had high frequencies of transfusion, invasive treatment, and early rebleeding. Right CDB seemed to be a risk for serious disease. Factors related to late rebleeding were different from those related to early rebleeding of CDB.

## 1. Introduction

The prevalence of colonic diverticula is increasing as the population ages in many countries, including Japan [1]. The number of patients with diverticular bleeding is rising in combination with the increasing use of antithrombotic and anti-inflammatory drugs. Colonic diverticular bleeding (CDB) is therefore becoming the main cause of lower gastrointestinal bleeding [2,3,4]. CDB is consequently a troublesome condition, because it occurs repeatedly and can sometimes be severe [5,6,7,8,9,10]. Recently, relevant academic societies published guidelines for treating CDB in Japan [11].

Reports to date have suggested that the use of nonsteroidal anti-inflammatory drugs (NSAIDs) and antithrombotic drugs, comorbidities, past diverticular bleeding, and physical findings such as shock are factors associated with exacerbation and rebleeding in CDB. However, the evidence remains weak, and predicting exacerbation and rebleeding in initial consultations is presently difficult [3,11,12,13,14]. Examining the predictability of associated factors using actual clinical data may therefore be of great significance.

Additionally, in this study, we focused on differences among clinical factors depending on the site of diverticular bleeding. The locations of colonic diverticula vary greatly by race and age. For example, colonic diverticula are mainly in the cecum, ascending colon, and right transverse colon in Asians, including the Japanese. Conversely in Caucasians, left colonic diverticula, mainly in the sigmoid colon, account for the majority of cases. In Japan, left colonic diverticula are more common in the elderly than in the young [1,15,16,17,18]. Diverticulitis, as another major complication of colonic diverticula, is known to have a higher rate of exacerbation in the left colon than in the right colon [19]. However, the site-specific clinical features of diverticular bleeding have not been sufficiently studied, and the amount of information on the condition is consequently small [20,21,22]. If the dependence of severity on the site of bleeding and differences in progression could be clarified, this could contribute to the selection of treatment methods and the prediction of prognoses during initial consultations.

This study therefore examined cases in which red blood cell (RBC) transfusion and invasive treatment were necessary, as well as predictors in cases of recurrence among patients with CDB. We also examined differences in the clinical features of diverticular bleeding between the right colon from the cecum to the transverse colon and the left colon from the descending colon to the rectum in confirmed cases of CDB where the site of bleeding was clear.

## 2. Materials and Methods

**Figure 1:**
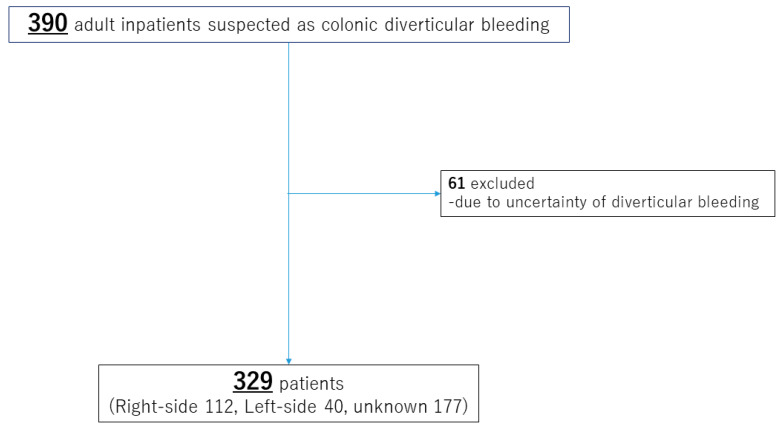
Flowchart of selecting subjects.

This study was a retrospective investigation of a single facility. The primary outcome of this study was to identify the factors related to the aggravation and recurrence of CDB in the patients. As the secondary outcome, we investigated the differences in clinical course according to the laterality of the bleeding site. Subjects were consecutive patients over 20 years old who had undergone inpatient treatment following a confirmed or suspected diagnosis of CDB (Figure 1).

**Figure 2:**
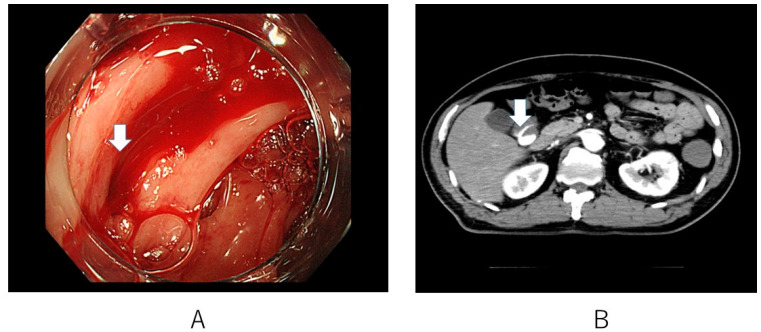
Definite colonic diverticular bleeding. (**A**) Bleeding from diverticula is observed with endoscopy (arrow). (**B**) Extraversation of the contrast medium into the colon lumen is demonstrated with computed tomography (arrow).

Patients were selected from among all the patients who underwent colonoscopy in the Department of Medicine at Teikyo University Hospital (Tokyo, Japan) between January 2004 and May 2021. In the case of patients with a history of multiple hospitalizations during the investigated period, only the first hospitalization was evaluated. Diverticular bleeding was diagnosed based on colonoscopy and/or CT findings. Confirmed cases were those in which: (1) bleeding from diverticula or exposed vessels was directly confirmed on endoscopy (Figure 2A); or (2) leakage of contrast medium in the colon was confirmed with contrast-enhanced CT (Figure 2B), and colonoscopy confirmed that gastrointestinal bleeding and diverticula existed and there were no other lesions that cause bleeding.

Suspected cases were those in which gastrointestinal bleeding was evident, and the presence of colonic diverticula was confirmed during endoscopic observation, no lesions other than the diverticula were apparent as potential causes of bleeding, and no flow of blood from the ileocecal valve to the colon was observed. In confirmed cases, the site of bleeding was judged to be the right colon in cases involving the cecum, ascending colon, or transverse colon, and to be the left colon in cases involving the descending colon, sigmoid colon, or rectum. Cases that did not fit the above definitions or in which CDB could not be diagnosed were excluded from being subjects. These judgments were independently made for each case by two endoscopists. When the two endoscopists were not in agreement, another endoscopist made the final judgment.

Each of the patients was investigated for background factors comprising physical data (age, height, and weight), smoking history, drinking history, comorbidities (hypertension, diabetes, dyslipidemia, ischemic heart disease, constipation, cerebral infarction, and history of diverticular bleeding), and concomitant medications (NSAIDs, antiplatelet drugs, and anticoagulants), as well as for information on diverticular bleeding, comprising information during bleeding (confirmed/suspected diagnosis, site of bleeding, blood pressure, heart rate, and hemoglobin level), treatment (endoscopic hemostasis, vascular embolization (IVR), surgical resection, and RBC transfusion), rebleeding, and survival. The occurrence or absence of rebleeding was evaluated and confirmed as either early rebleeding (occurring within one month of initial bleeding) or late rebleeding (occurring after one month but within one year of initial bleeding) based on medical records.

Statistical analyses were performed using SPSS version 28 software (IBM Japan, Tokyo, Japan). The Mann–Whitney U test and chi-squared test were used for comparison of the two groups. The effect of clinical factors was evaluated using a generalized linear model, after adjusting for background factors using the inverse probability of treatment, weighting with propensity scores calculated for each case. The level of statistical significance was 0.05 in all cases. The study protocol was approved by the ethics committee of Teikyo University before the study was started (approval no. TU 21-066). All procedures were performed in accordance with the relevant national and international guidelines, including the Declaration of Helsinki and its amendments. The need to obtain informed consent was waived by the ethics committee that approved the study, given the retrospective design of the study.

## 3. Results

### 3.1. Background Characteristics

A total of 329 patients was analyzed (Figure 1). Background data are presented in Table 1. Median age was 73 years, and approximately two-thirds (222, 67.5%) were men. Obese patients with a body mass index ≥ 30 kg/m^2^ only accounted for 18 patients (5.5%). A smoking history was seen in 95 (28.9%), and a drinking history was seen in 134 patients (40.7%). In addition, a high rate of patients (148, 45.0%) had a history of diverticular bleeding. In terms of comorbidities, 224 (68.1%) had hypertension, and 123 (37.4%) had dyslipidemia. Concomitant medications were anticoagulants in 53 (16.1%), antiplatelet drugs in 90 (27.4%), and NSAIDs in 66 (20.1%).

### 3.2. Data on Colonic Diverticular Bleeding

CDB was confirmed in 152 cases, including 112 cases with right colonic bleeding and 40 with left colonic bleeding. The remaining 177 cases had suspected CDB. Hemoglobin levels during bleeding were ≤10 g/dL in 146 cases (44.4%), and the shock index (heart rate/systolic blood pressure) was ≥1 in 19 cases (5.8%). As for treatment, 157 patients (47.7%) required RBC transfusion, 93 patients (28.3%) underwent endoscopic hemostasis, and a hemostatic clip was used in all patients. Interventional radiology (IVR) was performed in 13 cases (4.0%), and surgery was performed in 6 cases (1.8%). No cases of death during bleeding were seen. Early rebleeding within one month of initial bleeding occurred in 75 cases (22.8%), and late rebleeding within one year occurred in 62 cases (18.8%; Table 1).

Association of clinical factors with outcome

Table 2 shows the associations between RBC transfusion, invasive treatment, and rebleeding and clinical factors. Factors significantly associated with RBC transfusion were confirmed CDB, anticoagulants, high shock index during bleeding, and low hemoglobin level. The only factor associated with invasive treatment was confirmed CDB, and all patients who underwent surgery or IVR were confirmed cases. Factors significantly associated with early rebleeding were confirmed CDB and hypertension, while factors significantly associated with late rebleeding were hypertension, chronic kidney disease, and past CDB (Table 2). No significant associations were seen between NSAIDs and RBC transfusion, invasive treatment, or early or late rebleeding in this study.

The hemostatic effect of endoscopic treatment was not observed. Instead, early rebleeding tended to be more common in patients who underwent endoscopic hemostasis (adjusted odds ratio 7.590, 95% confidence interval [CI] 4.307–13.374, *p* < 0.001). Furthermore, no significant association was seen between late rebleeding and the performance or absence of endoscopic hemostasis (adjusted odds ratio 1.152, 95%CI 0.631–2.106, *p* = 0.645).

### 3.3. Difference between Right and Left CDB

Table 3 shows the differences in RBC transfusion, invasive treatment, and rate of rebleeding in right and left CDB. Median patient age was significantly lower for right CDB (71 years) than for left CDB (81 years) (*p* = 0.002, Mann–Whitney test). Patients with right CDB also tended to be male (*p* = 0.02, chi-squared test). Rates of patients who received RBC transfusion and underwent surgery or IVR were significantly higher for right CDB, but no significant differences were seen for early or late rebleeding between right and left CDB (Table 3).

## 4. Discussion

The main findings in this study were (1) confirmed CDB, concomitant use of antithrombotic drugs, shock upon hospital admission, and decreased hemoglobin were identified as factors associated with blood transfusion and invasive treatment; (2) confirmed cases of CDB showed a high rate of early rebleeding in addition to blood transfusion and invasive treatment; (3) patients with right CDB required RBC transfusion and invasive treatment more often than patients with left CDB; and (4) hypertension, chronic kidney disease, and past diverticular bleeding were risk factors for late rebleeding.

Contrast-enhanced computed tomography (CT) and colonoscopy are the main measures used to diagnose lower gastrointestinal bleeding, because contrast-enhanced CT is useful for identifying sites of bleeding and diagnosing underlying diseases [23,24], and colonoscopy allows hemostatic treatment as needed, in addition to high diagnostic performance [25,26,27]. All patients in this study had undergone endoscopy, and many had also undergone contrast-enhanced CT as well. Patients with confirmed CDB in whom bleeding was confirmed during the initial examination using these tests were assumed to have a considerable amount of bleeding at the time of or just before examination. This was likely associated with subsequent continuous or massive bleeding and may have resulted in a higher frequency of invasive treatment, RBC transfusion, and early rebleeding. Especially for invasive treatment, it is expected that treatment was not considered in unconfirmed cases, which may lead to selection bias. In other reports, continuous bleeding and early rebleeding in CDB are accompanied by a high risk of requiring RBC transfusion and invasive treatments such as surgery and arterial embolization [13,22]. Previously reported predictors included symptoms of shock, bright red blood in stool without diarrhea or abdominal pain, use of NSAIDs and/or aspirin, past bleeding, two or more comorbidities, and low hemoglobin level. However, these predictors alone were reportedly insufficient to predict present severity, so other predictors are required [11,12,13,14]. This study found the same associations with shock, antithrombotic drugs, and low hemoglobin level as previous reports, but invasive treatment, RBC transfusion, and early rebleeding also had significantly higher incidence in confirmed cases of CDB, suggesting their usefulness as new prognostic factors.

This study produced interesting results regarding the association between the site of bleeding and the severity. A significantly higher rate of patients with right CDB required RBC transfusion and invasive treatment compared with patients with left CDB. Kinjo et al. reported bleeding from diverticula in the right colon as a risk factor for severe diverticular bleeding, based on multivariate analysis, and suggested potential reasons, such as the following: right colonic diverticula are more susceptible to injury than left colonic diverticula because they are larger; the intestinal wall is thinner in the right colon than in the left colon and has relatively high blood flow; and bleeding is detected more slowly in the right colon because the distance from the anus is greater [20]. The causes of such differences were not evident in this study, but clinicians must keep in mind the high risk of requiring surgery or IVR in cases of bleeding from right colonic diverticula. CDB is a disease with a high rate of spontaneous hemostasis (73–88%) [5,6], and expecting spontaneous hemostasis with conservative treatment is valid in suspected cases where the site of bleeding has not been identified. However, active treatment is preferable from an early stage in confirmed cases of right CDB.

While endoscopic treatment is the predominant method of hemostasis, no inhibitory effect on early rebleeding was seen with endoscopic hemostasis in this study. Instead, rebleeding tended to be more common in the group that underwent endoscopic hemostasis. The cause of this unexpected result is not clear, since the details of endoscopic treatment have not been examined in the present study. Endoscopic hemostasis is generally performed in cases of severe disease or confirmed CDB with extravasation of contrast medium, which is a known situation in which rebleeding is common [28,29]. It is speculated that this result may be due to selection bias, as confirmed cases of CDB in the high-risk group for rebleeding underwent endoscopic hemostasis more often. Furthermore, these results may suggest that a hemostatic clip is not sufficiently effective, because a hemostatic clip was applied for hemostasis in all the cases. One of the countermeasures mentioned is selecting an endoscopic hemostatic method such as one using endoscopic band ligation (EBL) or a snare, as such methods have recently been reported to be effective [30,31,32,33,34,35,36,37,38,39,40]. According to a recent meta-analysis, rebleeding occurred significantly more with hemoclip than with EBL [41]. If the stigmata of recent hemorrhage (SRH) is at the bottom of the diverticulum or at the small orifice of the diverticulum, it is too difficult to clip the SRH directly, and inadequate clipping may lead to rebleeding. Niikura et al. reported that performing endoscopic hemostasis within 24 h, hemostasis performed by a specialist endoscopist, and use of a tip hood or water jet device, among other approaches, are useful in identifying the site of bleeding and could benefit future hemostatic procedures [42]. However, irrespective of the method of endoscopic treatment applied, late hemostatic effects cannot be expected, and the limitations of endoscopic treatment itself must be acknowledged [43].

In this study, late rebleeding within one year was seen in 18.8% of cases. Previous reports have described similar or slightly higher rates of rebleeding within one year, ranging from 20% to 35% [6,9,10]. The difference may be due to the retrospective study design of this study. Among the cases of rebleeding within one year, cases of visits to other facilities are not counted, and the possibility that the incidence rate has decreased cannot be ruled out. Hypertension, chronic kidney disease, past CDB, and other comorbidities associated with arteriosclerosis have been suggested to be significant risk factors. Furthermore, because risk factors for late rebleeding differed significantly from those in the acute phase and exacerbation, particular caution must be taken with patients with the above-mentioned risks during follow-up for bleeding. However, we believe that the details of risk factors for rebleeding should be reassessed in a prospective multicenter review to be precise.

The limitations of this study included its implementation in a single facility, the small number of subjects, and the retrospective study design. In particular, the influence of sample size seemed to be large. Although there were statistically significant differences in the relationship between shock and RBC transfusion, and between bleeding site and invasive treatment, the 95% CI was very wide. In order to improve the reliability of the results, it is essential to consider increasing the number of subjects. The entry period was also long, and medical advances and changes to treatment made during this period may have impacted the results. Furthermore, it is desirable to consider a prospective design in order to confirm the details of accurate factors affecting the prognosis of CDB, because retrospective cohort assessment of incidence is limited. It is actually hard to separately inquire into the different bleeding situations in the different kinds of CDB, especially in a retrospective study. A prospective study involving multiple facilities is required in the future, if feasible.

## 5. Conclusions

The present study showed that confirmed CDB had high frequencies of transfusion, invasive treatment, and early rebleeding. Right CDB seemed to be a risk factor for serious disease. Factors related to late rebleeding included hypertension, chronic kidney disease, and past CBD, indicating difference from factors related to early rebleeding of CDB.

## Figures and Tables

**Table 1:** Background characteristics of the subjects.

Characteristics	
Number of patients	329
Age (year), median (min-max)	73 (35–98)
Age >= 75 years old, n (%)	145
Sex (male), n (%)	222 (67.5)
Height (m), median (IQR)	1.62 (1.53–1.68)
Weight (kg), median (IQR)	59.9 (50.8–67.8)
Body mass index (kg/m^2^) > 30, n (%)	18 (5.5)
Smoking, n (%)	95 (28.9)
Alcohol, n (%)	134 (40.7)
Comorbidities, n (%)	
Hypertension	224 (68.1)
Dyslipidemia	123 (37.4)
Diabetes mellitus	63 (19.1)
Constipation	52 (15.8)
Ischemic heart disease	69 (21.0)
Cerebrovascular disease	43 (13.1)
Chronic kidney disease	166 (50.5)
Past diverticular bleeding	148 (45.0)
Concomitant medicines, n (%)	
Antiplatelets	143 (43.5)
Anticoagulants	53 (16.1)
Nonsteroidal anti-inflammatory drug	66 (20.1)
Diverticular bleeding	
Location of bleeding diverticula (right-left-unknown)	112-40-177
Hemoglobin < 10 g/dL, n (%)	146 (44.4)
Heart rate/systolic blood pressure > 1, n (%)	19 (5.8)
Treatment	
Endoscopic hemostasis	93 (28.3)
Surgery	6 (1.8)
Interventional radiology	13 (4.0)
Blood transfusion	157 (47.7)
Rebleeding	
Early (within 1 month)	75 (22.8)
Late (within 1 year)	62 (18.8)

Abbreviations: IQR—interquantile range; n—number.

**Table 2:** Impact of clinical factors on blood transfusion, invasive treatment, and rebleeding in patients with diverticular bleeding.

Factors	Red Blood Cell Transfusion, n; 157	Surgery and/or IVR, n; 19	Rebleeding within 1 Month, n; 75	Rebleeding within 1 Year, n; 62
Older age (≥75) n; 145	78(54) 1.63(0.71–3.76) *p* = 0.25	6(4) 0.34(0.11–1.04) *p* = 0.06	27(19) 1.21(0.34–4.33) *p* = 0.77	33(23) 0.79(0.34–1.84) *p* = 0.59
Hypertension, n; 224	115(51) 1.39(0.73–2.68) *p* = 0.32	13(6) 0.82(0.25–2.72) *p* = 0.75	57(25) 3.16(1.62–6.18) *p* < 0.001	49(22) 2.63(1.22–5.67) *p* = 0.01
Chronic kidney diseasen; 166	83(50) 0.81(0.48–1.36) *p* = 0.42	9(5) 0.90(0.34–2.37) *p* = 0.83	36(22) 0.85(0.46–1.54) *p* = 0.58	43(26) 2.79(1.44–5.40) *p* < 0.01
Past DBn; 148	59(40)0.66(0.42–1.06) *p* = 0.08	10(7) 1.44(0.55–3.77) *p* = 0.46	33(22) 1.070(0.62–1.84) *p* = 0.81	39(26) 2.10(1.13–3.85) *p* = 0.02
Antiplatelets, n; 90	52(58) 1.05(0.42–2.64) *p* = 0.92	8(9) 2.97(0.60–14.83) *p* = 0.18	23(26) 2.00(0.74–5.38) *p* = 0.17	15(17) 0.43(0.15–1.21) *p* = 0.11
Anticoagulants n; 53	34(64) 2.23(1.21–4.10) *p* = 0.01	5(9) 1.95(0.67–5.66) *p* = 0.22	16(11) 1.590(0.83–3.06) *p* = 0.16	11(21) 1.155(0.56–2.40) *p* = 0.70
NSAIDs n; 66	24(36) 0.32(0.39–1.37) *p* = 0.73	1(2) 0.26(0.03–1.99) *p* = 0.19	12(18) 0.74(0.36–1.53) *p* = 0.42	16(24) 1.26(0.62–2.53) *p* = 0.52
Confirmed DB n; 152	96(63) 2.88(1.77–4.69) *p* < 0.001	19(13) N/A *	57(38) 6.19(3.34–11.48) *p* < 0.001	28(18) 1.27(0.71–2.28) *p* = 0.42
Hb < 10 g/dL n; 146	102(70) 5.40(3.36–8.67) *p* < 0.001	7(5) 0.72(0.28–1.87) *p* = 0.50	35(24) 1.13(0.67–1.89) *p* = 0.65	33(23) 1.55(0.89–2.70) *p* = 0.12
HR/sBP > 1 n; 19	18(95) 22.1(2.9–168.0) *p* < 0.01	19(100) N/A *	7(37) 2.08(0.79–5.48) *p* = 0.14	1(5) 0.23(0.03–1.73) *p* = 0.15

*: Odds ratios are not available because all the cases that received surgery and/or IVR showed definite DB and HR/sBP above 1. Values express adjusted number (%) and odds ratios (95% confidence interval), which were evaluated with a generalized linear estimating equation model using a propensity score (older elderly (75 years or more), sex, body mass index more than 30, alcohol, smoking, hypertension, diabetes mellitus, dyslipidemia, chronic heart failure, ischemic heart disease, chronic kidney disease, cerebral vascular disease, past history of colonic diverticular bleeding, constipation, nonsteroidal anti-inflammatory drugs, antiplatelets, anticoagulants, detection of bleeding site, hemoglobin below 10 g/dL, heart rate/systolic blood pressure < 1), weighting by the inverse probability weighting method. Abbreviations: IVR—interventional radiology, DB—diverticular bleeding, NSAIDs—non-steroidal anti-inflammatory drugs, Hb—hemoglobin level, sBP—systolic blood pressure, HR—heart rate.

**Table 3:** Clinical differences between right-sided and left-sided diverticular bleeding.

Characteristics	Right-Sided 112 Patients n (%)	Left-Sided 40 Patients n (%)	Adjusted Odds Ratio (95% CI)	*p*-Value
Red blood cell transfusion	76 (67.9)	20 (50.0)	3.38 (1.36–8.38)	0.009
Invasive treatment (IVR or surgery)	18 (16.1)	1 (2.5)	27.49 (3.46–218.51)	0.002
Early rebleeding (within 1 month)	44 (39.3)	13 (32.5)	0.85 (0.31–2.31)	0.747
Late rebleeding (within 1 year)	18 (16.1)	10 (25.0)	0.90 (0.32–2.51)	0.843

Adjusted odds ratios were evaluated with a generalized linear estimating equation model using a propensity score (older elderly (75 years or more), sex, body mass index more than 30, alcohol, smoking, hypertension, diabetes mellitus, dyslipidemia, chronic heart failure, ischemic heart disease, chronic kidney disease, cerebral vascular disease, past history of colonic diverticular bleeding, constipation, nonsteroidal anti-inflammatory drugs, antiplatelets, anticoagulants, detection of bleeding site, hemoglobin below 10 g/dL, heart rate/systolic blood pressure > 1), weighting by the inverse probability weighting method. Abbreviations: CI—confidence interval, IVR—interventional radiology.

## Data Availability

The data supporting the findings of this study are available on request from the corresponding author, Takatsugu Yamamoto (ymmt@med.teikyo-u.ac.jp) at Teikyo University School of Medicine. The data are not publicly available due to the inclusion of information that could compromise the privacy of research participants.
